# Supplementary material for: Black kites of different age and sex show similar avoidance responses to wind turbines during migration
Source: R Soc Open Sci. 2021 Jan 20;8(1):201933. doi: 10.1098/rsos.201933 (PMC7890477; doi:10.1098/rsos.201933)
Supplement: Supplementary information [file rsos201933supp1.pdf]

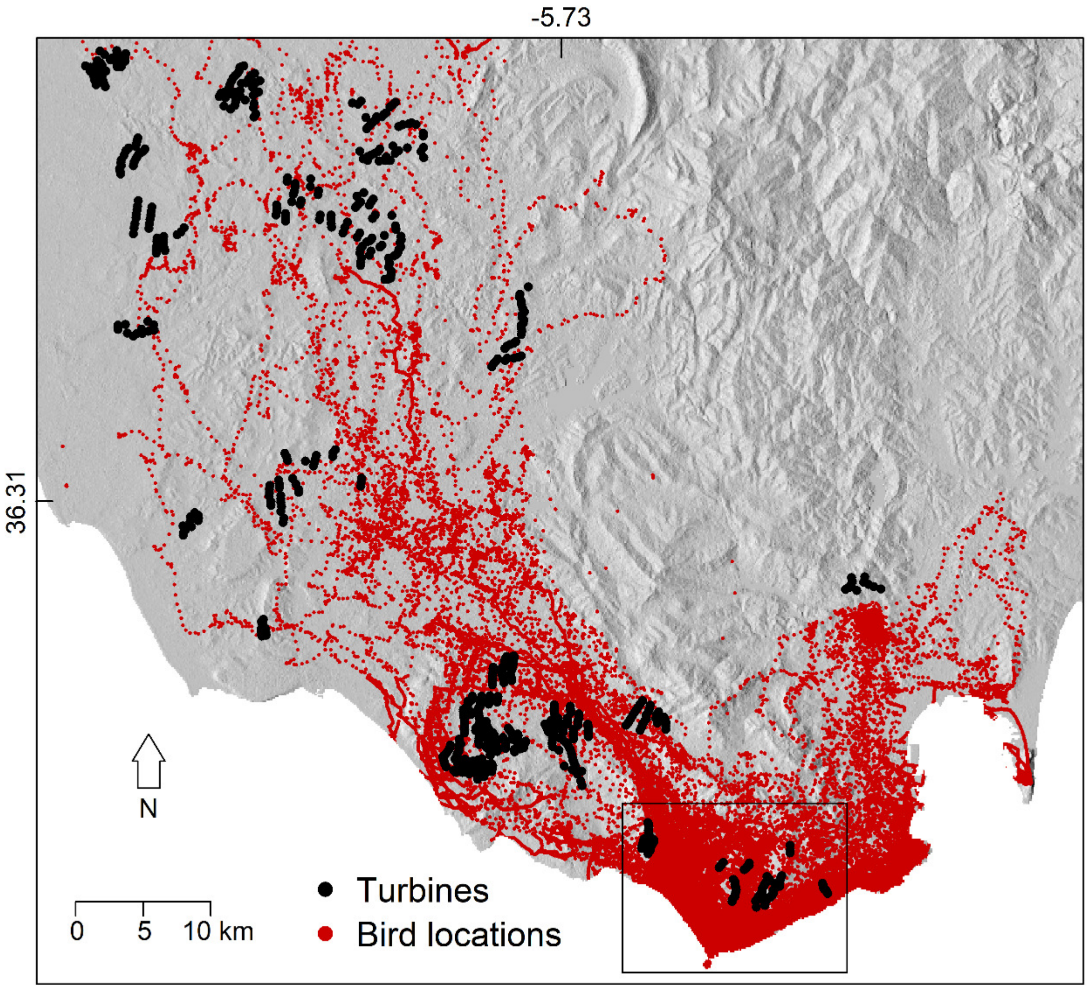

Figure S1. Spatial distribution of the full dataset and the location of the area selected for modelling purposes (marked with a rectangle).

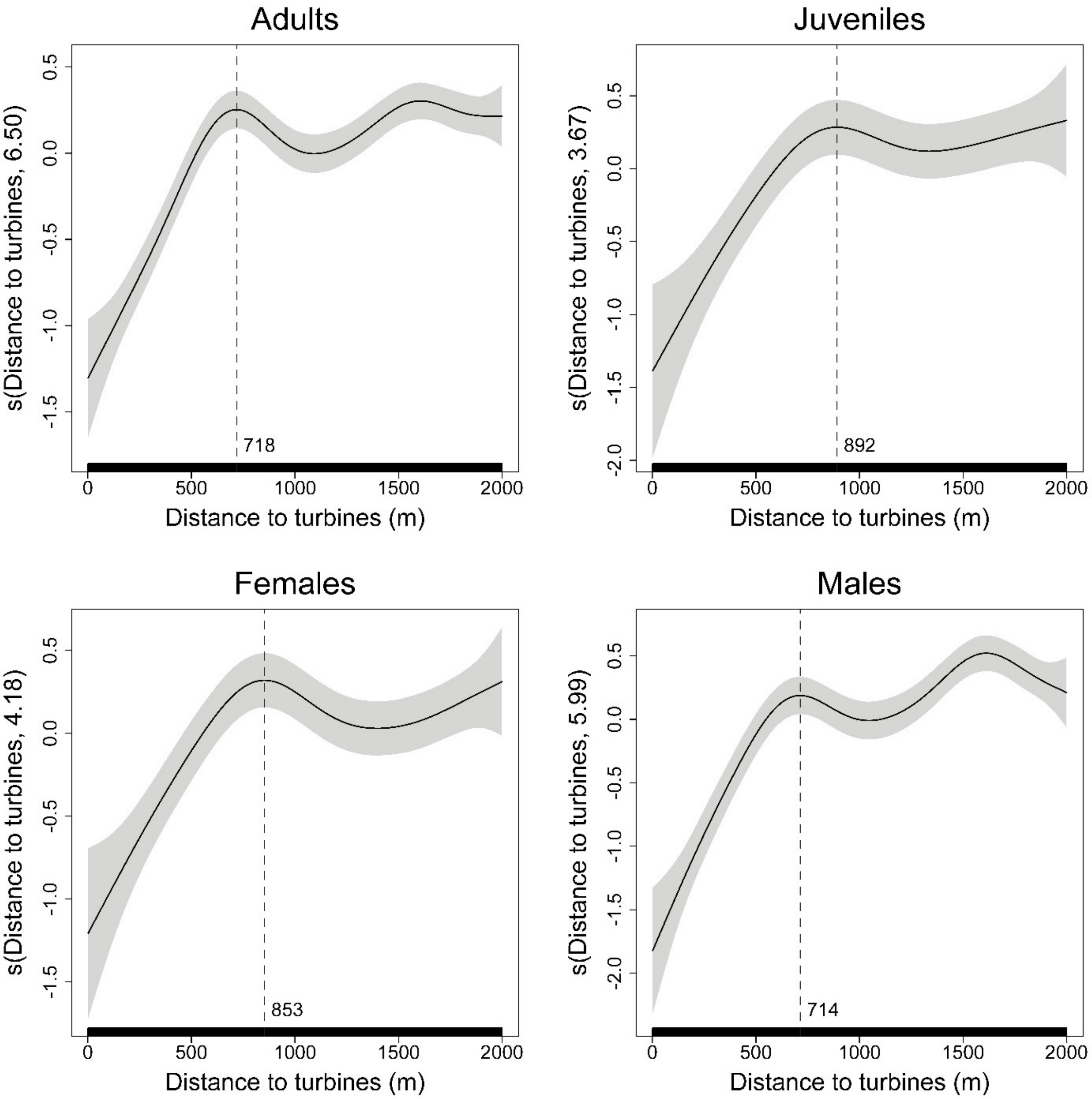

Figure S2. GAMM partial effects of turbine proximity on utilization distribution (UD) of black kites. In these models, we did not exclude extreme UD values as for those shown in figure 2 (see methods). Different models were built for each sex and age class. All four models included orographic and thermal uplift velocities as predictors, and accounted for spatial autocorrelation. Shaded areas represent 95% confidence intervals.

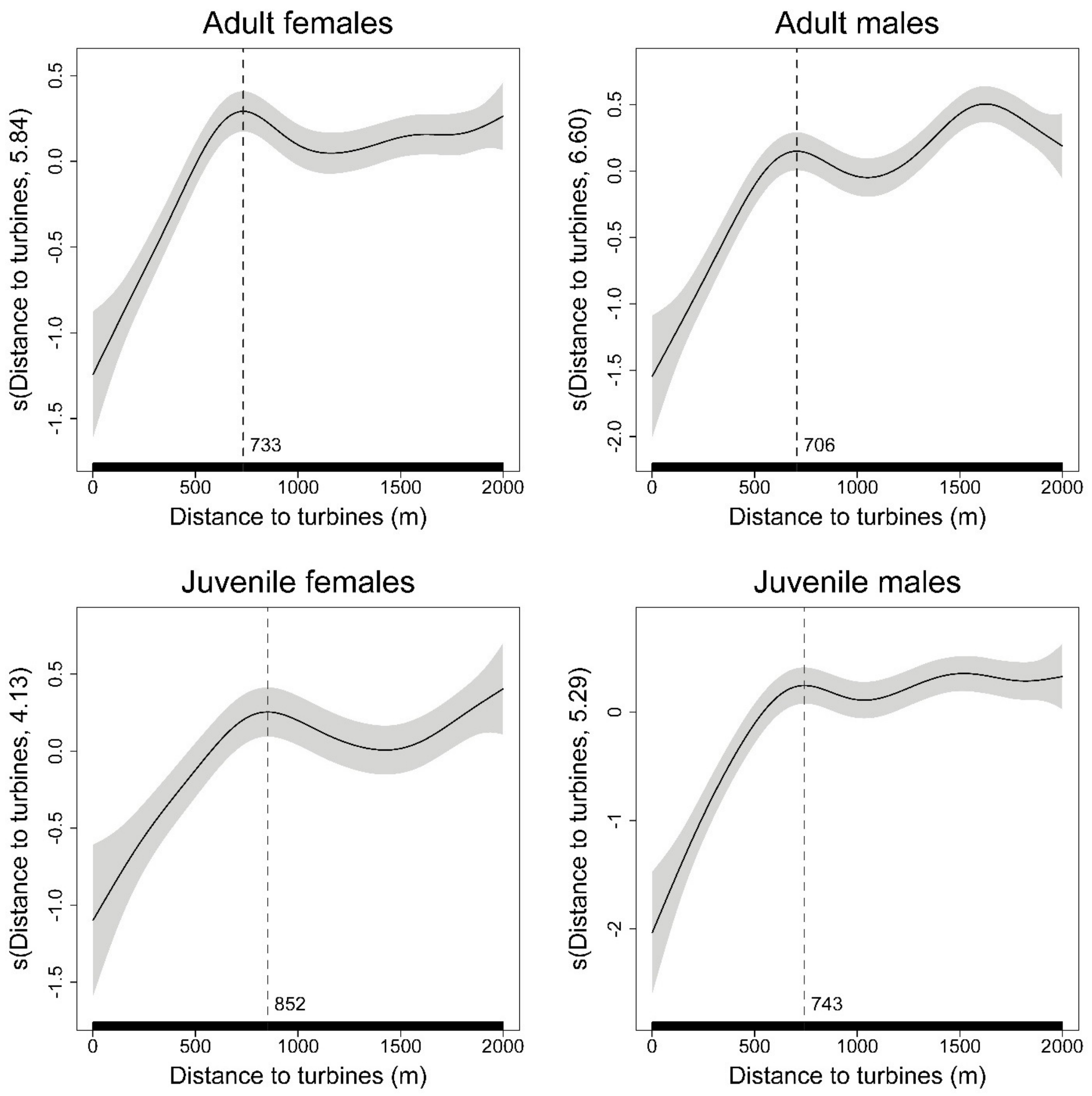

Figure S3. GAMM partial effects of turbine proximity on utilization distribution (UD) of black kites. Different models were built for each group. All four models included orographic and thermal uplift velocities as predictors, and accounted for spatial autocorrelation. Shaded areas represent 95% confidence intervals.

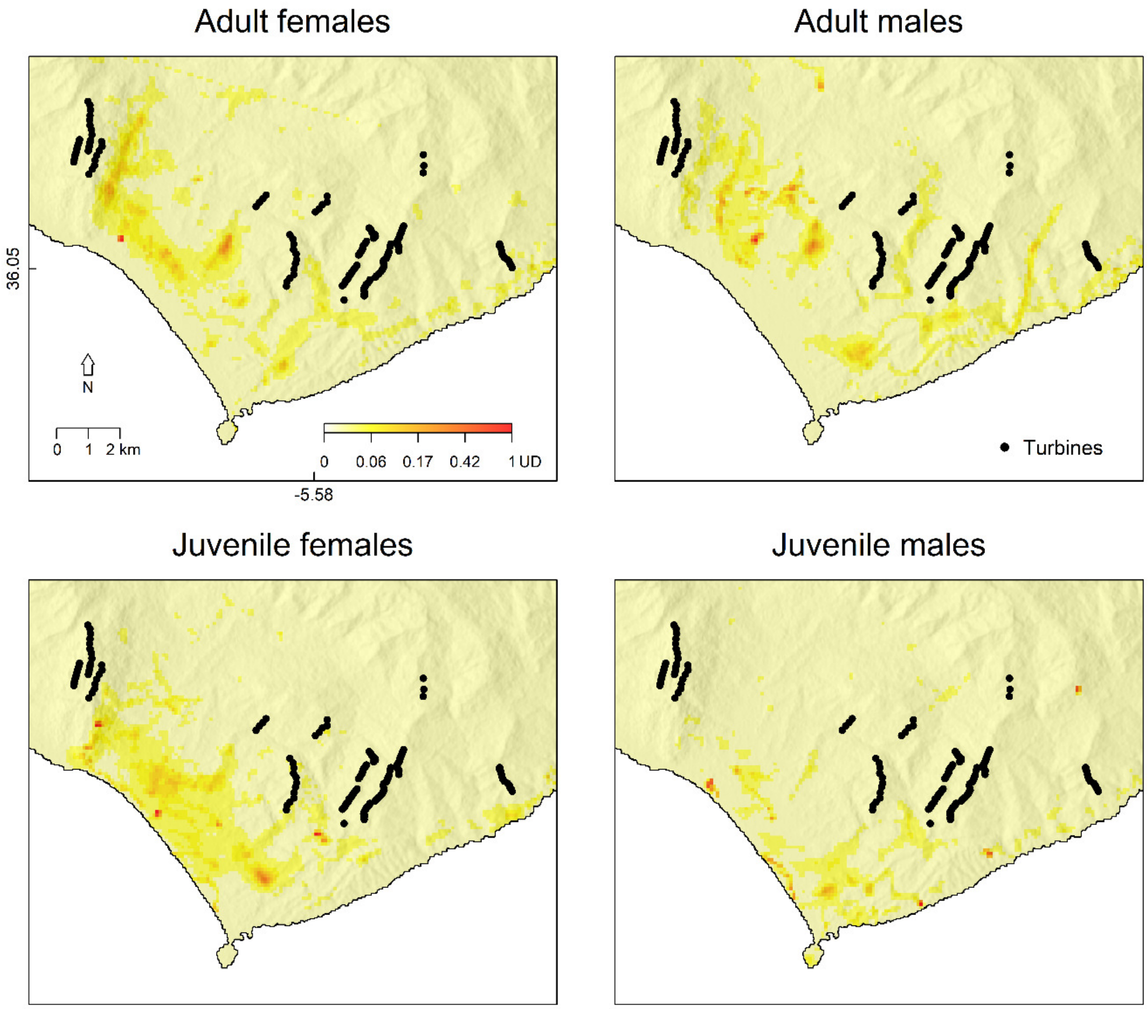

Figure S4. Utilization distribution (UD) of black kites in the study area (Tarifa, Spain) discriminated by sex and age group combinations. UD values are shown in a colour gradient, with darker colours reflecting higher UD. All plots follow the UD scale presented in top left plot. UD values result from dBBMMs build with GPS tracking data of 135 birds caught during the post-breeding migration in 2012 and 2013. UD resolution is 100 x 100 m. Hill shading was added to image background to show interaction between bird movement and topography.

R scripts for the main steps of data analysis

Building up the DBBMMs

#Uses a dataframe with Time, Easting and Northing UTM coordinates of each GPS fix. Creates a raster object with the DBBMM result. This step was repeated for data of each bird in each day. The raster objects were summed up for each category compared, i.e., all juveniles, all adults, all males, or all females. Note that the input dataset is produced from the dataset made available in Movebank after several steps of filtering, detailed in the methods. Filtering steps selected birds that were in flight (with speed > 1 m/s), recorded in a target area (shown in figure 1) and during easterly winds (wind direction 70 to 130º).

```
#Creates a move object out of the dataframe with tracking data
library(move)
dataset_move<- move(x=dataset$EastingUTM, y= dataset$NorthingUTM,
time=as.POSIXct(dataset$Time, format="%Y-%m-%d %H:%M:%S"), proj=CRS("+proj=utm
+zone=30 +datum=WGS84 +units=m +no_defs +ellps=WGS84 +towgs84=0,0,0"))
#Builds the DBBMM. Uses a raster object covering the range of the full dataset, named raster_base
DBBMM <-brownian.bridge.dyn(dataset_move, raster= raster_base, location.error = 20,
window.size=15,margin=5)
# Sums up DBBMMs per category.
DBBMM_juveniles<- DBBMM_ind1_day1+ DBBMM_ind2_day2+DBBMM_ind2_day1…
DBBMM_adults<- DBBMM_ind3_day1+ DBBMM_ind3_day2+DBBMM_ind7_day1…
```

Building up the GAMMs

#Uses a dataframe with data from each cell of the 100x100 m grid covering the study area. The data includes Easting and Northing UTM coordinates, UD extracted from DBBMM raster objects, distance to the closest wind turbine, and orographic and thermal uplift obtained as detailed in the methods. Different GAMMs were fit with datasets of each category, i.e., juveniles, adults, males or females.

```
library(mgcv)
model_juveniles<-gamm(juvelines_UD~s(turbine_distance)+ thermal_uplift + orographic_uplift
,correlation=corGaus(form=~ EastingUTM+NorthingUTM),family=Tweedie(1.6,power(0)),
data=dataset)
```
